# Observations in Practical Surgery: On the Use of Water by the Method of Affusion; on the Infrequent Dressing of Wounds, &c.

**Published:** 1837-01

**Authors:** 


### ART. VI.

*Mélanges de Chirurgie Pratique: Emploi de l'Eau par la Methode des Affusions; Pansemens rares, &c. D'après la Clinique Chirurgicale de l'Hôtel-Dieu d'Amiens et les Leçons de* M. JOSSE, Chirurgien en chef de l'Hôtel-Dieu d'Amiens, &c. Par M. JOSSE, Fils, Chirurgien aide de l'Hôtel Dieu, &c.—*Paris,* 1835. 8vo. pp. 366.

*Observations in Practical Surgery: On the Use of Water by the Method of Affusion; on the infrequent Dressing of Wounds, &c. According to the Practice of* M. JOSSE, Chief Surgeon of the Hôtel Dieu of Amiens, &c. By M. JOSSE, Jun., M.D., Assistant Surgeon of the Hospital, &c.

ON examining the contents of the present volume, we were led to the conclusion that, as its scope is mainly practical; as it embraces the result of very considerable experience; as the author's observations are often acute and sagacious; and as the subjects treated are of very great importance,—a review of it might be both acceptable and beneficial.

M. Josse, the author, is a surgeon residing in the large provincial city of Amiens, and attached to its hospital, which is of considerable size and consequence. His father has long been at the head of the surgical department, and the modes of practice which are here advocated appear to have chiefly originated with him, though the son, with commendable regard, has not only adopted them, but illustrated them with zeal and talent. In treating of this work, we can make no distinction between the two; but it will be understood that the cases were chiefly under the care of the father.

A preliminary dissertation embodies the author's opinions on the nature of inflammation, and in it we see nothing so new or so precise as to entitle it to particular notice; we shall therefore content ourselves with saying, that the leading doctrine he adopts is, that the heat of the inflamed part is more amenable to treatment than any other phenomenon belonging to that process, and therefore "it is against it the efforts of art should be directed," (p. 30;) and on this principle he bases his practice; we will not here stop to enquire how correctly; for, after all, the question is not so much, whether he is right in this opinion, or whether it is good to the extent proposed in the present essay. When practice is founded on theory, it is necessary strictly to examine whether that theory is correct, when, on experience, the case is different; facts are facts, and we have only to endeavour to ascertain whether they are correctly stated, and whether the deductions from them are legitimate.

Cold water is the almost universal means the author proposes for the purpose of combating the symptom above alluded to, and the following is his own account of the mode in which he recommends its application.

"If we had always the choice," he says, "it might be established as a general principle that we ought to employ water by *affusion with a continual stream;* but the nature of the parts or of the disease may prevent this, and oblige us to recur to another method; thus, linen moistened with water, and renewed without ceasing, may to a certain degree prove a substitute for the affusions, but this mode requires much attention: the compresses should be applied lightly, or rather simply laid on, so that the air passing across the folds may carry off the vapour produced by the morbid heat and a fresh stratum of cold water may in turn replace that which has been evaporated, and so absorb a new quantity of caloric as before." (P. 32.)

By these means a great degree of cold may be produced, "but it is necessary that the compresses should be applied lightly, (*negligemment,*) and leave numerous spaces for the free passage of the air, to carry off the vapour it becomes charged with." This mode of applying cold, he says, is well adapted to those cases in which affusions cannot properly be employed; as "when injuries are situated in certain regions of the trunk or face, or when, the symptoms being very feeble, there is reason to apprehend that too brisk an application of cold might do more harm than good." (P. 33.) In the limbs, he says, affusions are always possible, and are indispensable in cases of inflammation already severe or likely to become so. The following is his own method of using them.

"A vessel with a cock near its base,* is filled with water, and placed upon a narrow and high table, near the patient's bed, in such a position that it shall be about a foot and a half above the diseased limb, beneath which a cerecloth is spread, intended to guard the bed, and facilitate the flow of the water, which is received in a bucket placed near for that purpose and into which the extremity of the cerecloth descends."

In order to change the current more readily, it is a good plan to adapt a tube of elastic gum to the cock, or other aperture of the vessel from

* Probably a common tea-urn would answer this purpose well.—Rev.

which the water is poured. Other guards for the bed may be devised either of skin, metal, or other substance; but it is well to arrange them so, that a gutter shall be formed in which the limb may be received; a plan, especially in fractures, useful in keeping the parts steady, and also in carrying off the water more readily.

"Every thing being prepared, the diseased part should be placed in the most convenient position; it should be lightly covered with compresses; an additional piece of linen should surround the cock by one of its extremities, while the other is extended over the highest point of the apparatus. This is destined to prevent the water from falling with all its weight on the diseased part, and rather to disperse it over a larger surface . . . . The affusion ought to be made over the whole disease at once, otherwise it (the inflammation) gains in some parts, while it yields in others, where the affusion does not reach." (P. 34.)

The objection to mere moistened compresses is, as he says, that they soon acquire the temperature of the part, and that there is a kind of perpetual oscillation of heat and cold from their renewal, (p. 38, 47); but, while this must be admitted, it is also clear that a remedy may be found in many cases, by renewing them very frequently, by dropping water on them from a sponge (an imperfect affusion), by passing a current of air over them, or by other devices; we, however, readily allow that his mode of applying water by affusion is sufficiently simple, and very effective in severe cases. It is, as we stated in our last number, by no means unknown or unemployed in our hospitals in this country.

"The temperature of the water should be less than that of the parts to which it is applied, and as low as the patient can bear without pain," (p. 40.) It may produce one of these effects: "1st. It may absorb little heat and produce little relief; sometimes even the pain increases under its use. 2dly. It will produce a painful degree of cold, and, if its action is not suspended, the sufferings, though from a different cause, may become as severe as those it was meant to prevent." 3dly. It will give unqualified relief, (p. 41-2.) In the first case, the temperature should be reduced by ice or other means; in the second, it should be raised. He also maintains that the effect produced does not merely depend upon its influence on the temperature, but also on the due quantity of water being brought into contact with the inflamed part, in consequence of a certain relation between the disease and the remedy, which he hints may be owing to some electrical influence.

M. Josse admits the frequent repugnance of patients to the use of cold water; the unpleasant feelings it occasionally produces; the length of time before it exerts a beneficial influence on deep-seated inflammations, although that is not less ultimately: also, the want of faith in patients as to the efficacy of so simple an application; nevertheless, he is not disposed to yield either to the prejudices of the patient (or, it may be, to the advantage of his attendant,) the privilege of disguising it by any medication.

The author hardly enters into the question of this mode of treatment being likely to produce internal inflammations; he disposes of it in the negative, in a brief note, (p. 44.) Nevertheless, this point cannot be altogether overlooked, but undoubtedly demands more attention than it has hitherto received: in scarlatina, for instance, as is well known, it is commonly highly beneficial, and has little tendency to produce metastasis; in rubeola it is quite the reverse; in some cases of gout it has been employed with impunity; in others, its results have been fatal: in erysipelas accompanied with great heat and tension, it may be used fearlessly; but when the actions are feeble it undoubtedly occasions, and that not unfrequently, very serious increase of the constitutional symptoms, and formidable affections of internal organs: in injuries from accident, it is not often that it produces ill effects, but still cases do occur, not where the inflammation is repelled, (for that supposes spontaneous origin,) but where the patient catches cold, as it is called, and gets an attack of pulmonary or other internal inflammation.

M. Josse proceeds to particularize the forms of inflammation in which he states from experience that cold water may be employed with success; and this he does in chapters as follow: namely, on Erysipelas, Phlegmon, Burns, Contused Wounds, and on Gangrene. He has also a chapter on the opening of Abscesses, (the third,) and another (the sixth) on Dressing Wounds, which as they do not appear to be very advantageously introduced in their present order, we shall consider apart.

*Erysipelas.* M. Josse gives two cases occurring on the face and head, and two in the extremities, treated with cold affusions; the latter, being of the kind denominated E. Phlegmonodes, deserve to be mentioned from the circumstances under which the cold was applied and continued with success: they are briefly as follows:

"Case iii. Woman, æt. forty-five, admitted five days after the attack. The disease extended over the leg, knee, and outside of the thigh; there was great swelling; the skin tight, shining, bright red at some points, at others violet; many large phlyctenæ, of which several were broken, with black gangrenous spots below: some mortified spots on the outside of the thigh, no fluctuation, but extensive doughiness, ('*empâtement*') *heat* excessive; pains very severe, pulsative; violent fever; all the symptoms high. Cold *affusion* energetically employed. The following day, the adjacent parts much improved, and progress of the disease arrested; the parts where the erysipelas was in an advanced stage better; fever greatly reduced. Third and fourth days further improvement; and, at the end of the twelfth, the patient went out perfectly cured without any abscess." (P. 59.)

In this case there was no other treatment mentioned.

"Case iv. Woman, æt. forty-nine, received several severe contusions on the middle and outside of the forearm. Erysipelas ensued. Admitted into the hospital seven days after the accident. The limb then in an advanced stage of erysipelas, with '*empâtement*,' crepitation, burning heat, severe and high pyrexia: no hope of preventing suppuration and gangrene. Cold applied. Following day, the disease had rather made progress. Twenty leeches applied to the shoulder. Next day, (December 17th,) neck and chest invaded: sphacelus of the inside of the arm. Pulse sharp, concentrated; vomiting, diarrhœa, cold sweats. It was found that the patient had interfered with the efficient application of the cold. The affusion was now energetically applied over the whole surface of the arm. The following day, the erysipelas was stopt, and the swelling of the shoulder had subsided. Abscesses of the forearm were opened by multiplied short incisions; enormous discharge, and of bad quality; sloughs loosen; almost all the skin of the forearm insulated; general symptoms abate. 19th. Further improvement. 21st and 22d. Patient still better. Cold affusion continued with slight pressure. Fall of slough on forearm. Constitutional affection at an end. Water begins to be unpleasant and was stopt. 24th. Renewal of symptoms; cold resumed; impression painful; water applied tepid, which agreed perfectly. In short, the patient got well in six weeks without any deformity of the limb." (P. 62.)

These cases shew that cold may be very efficacious in the phlegmonoid form of erysipelas even in the *advanced* stages; and from no other curative measures being adopted, except local means of partial effect in the second, the benefit here is not imputable to any other cause; but, while we freely recognize the great utility of cold applications in this species, especially in the early periods, and even think its advantages may be increased in many instances by sponging the whole body when the weather is hot and the fever high, as well as using it locally; we cannot think it will exclude other means of great efficacy which it is unnecessary to detail here, nor exempt us from the necessity of attending to the constitutional symptoms, which M. Josse intimates will be subdued with the local disease. To other measures it is a very powerful auxiliary; and, to give it full effect, we are quite disposed to agree that M. Josse's plan of employing it is the best. With respect to erysipelas of a different character, we cannot but say that we consider the apprehensions generally entertained of the danger of repulsion not unfounded, although they are altogether disregarded by our author, nor do we hold in such little estimation as he does the application of the argenti nitras, mercurial ointment, or various forms of diluted alcohol.

*Phlegmon.* Under this head M. Josse includes not only common phlegmon, but cellular and thecal inflammation, &c. This is much to be regretted, as, without more precise distinction, it is hardly possible to know how far we are reasoning with accuracy: we must therefore consider his argument in this chapter as too general for close criticism. His opinions are briefly these: phlegmonous inflammation has a great disposition to suppurate, and hence it is very generally considered that it is useless to endeavour to resolve it: this opinion he combats, and proceeds to examine the various modes by which resolution may be attempted. Bleeding, he says, only calms the general symptoms: leeches aggravate the complaint. Incisions are only proper in extreme cases. Poultices he strongly condemns, with a few exceptions; he states that, far from being emollient, as they are generally considered to be, in point of fact, they augment the actions of the part, the swelling, and the pain; and, even when suppuration occurs, they increase the suppurative action, and thereby do harm. If he makes an exception, it is in favour of cold poultices while they continue cold, and thereby act in the same way as cold water. His opinions on this point are embodied in the following positions, laid down at p. 79 et seq.

"1st. When inflammation occupies the skin only, or the skin participates more or less in the irritation of subjacent parts, poultices will be always injurious." "2d. When the irritation is situated beneath the skin more or less deeply, and the skin is healthy, poultices will be of great use." "If you wish to obtain resolution of inflammation," he says, "abandon poultices in phlegmon, erysipelas, contusions, burns, contused wounds, &c.; employ them in deep abscesses, pleurodyne, arthritis, neuralgia, &c.; always when you wish a derivation."

There are objections to all other remedies for phlegmon but "cold water alone, which unites every advantage of each of the others without having their inconveniences."

With such sweeping censure of poultices, and unbounded commendation of cold water, we are not prepared to deal, for the reasons above stated: they may be perfectly well-founded as regards one species of inflammation, as for instance thecal, and mainly erroneous in another, as in that form of limited inflammation, which is perhaps correctly termed a phlegmon. The valuable and objectionable practice in this part of the work of M. Josse's is so much blended, that it is difficult to say whether we are more disposed to approve or condemn; and, while we can by no means admit that, according to his axiom, poultices are to be abandoned in all cases where *the skin is inflamed*, we agree with him in thinking that cold is greatly preferable in those common (and as far as the limbs are concerned) very fatal cases of inflammation, the thecal and fascial, not only during their early progress, but when matter has formed, and openings have occurred. The cases he has given are well calculated to establish the utility of an energetic use of the affusion in these, but we entirely dissent from his expressed opinion as to the inferior utility of incisions.

*Burns.* To every degree of injury from heat, from the simplest scald to the most severe burn, without regard to its extent, without exception of age or constitution, from the commencement to the termination, M. Josse would apply one remedy, cold water, (p. 117.) There is no other form of application which he does not condemn, either as being directly injurious, or as an imperfect modification of his favorite remedy. If alcohol or æther serve, it is merely by the cold they produce; the poultices of scraped potatoes have no other effect; and to cotton only he allows some utility in burns which must suppurate. Mr. Kentish's plan he compares to the works of the darker ages, when wounds were cauterized with boiling oil. We cannot be expected to examine minutely the charges thus brought against many well-known forms of treatment, which have, after a careful comparison with that by cold, received the sanction of the best surgeons in this country. Where cold can be efficiently applied for a long time; when the extent of the mischief is not very great; where the constitution of the patient affords no impediment; we believe it to be a very useful and a very grateful application: but even where all these circumstances combine to render it proper, there are many which may make it inconvenient. To be effectual, it must be continued night and day, which involves the necessity of a constant attendant by night, on the one hand, and more or less interruption to the patient's repose, on the other: in severe cases such considerations are not to be regarded; but in many of these, this mode of treatment, on various accounts (alluded to above,) may be inexpedient, and in slight cases the plan will be imperfectly submitted to.

M. Josse condemns Mr. Kentish's plan, it would seem, because he cannot reconcile the principle on which it acts, (p. 116.) We will not discuss this matter; it is often better to deal merely with facts, and with reference to this there is hardly an hospital in Great Britain which will not produce many in favour of this condemned practice. Since the introduction of the treatment by flour and cotton, we have seen more benefit derived from these, particularly the former, than from any other mode of treatment. From circumstances, however, which at present it seems hard to determine, of the many modes proposed, some one may have the advantage in particular cases. Although we by no means concur with the author in this doctrine of the universal superiority of cold water, we are bound to add, that some of his observations are judicious: thus, there appears to be much reason in his objection to the not uncommon opinion that it is right to treat these injuries according to the *degree* of heat which has produced them; that in severe cases every degree exists in some part of them, (p. 111;) that the best and most expeditious mode of accelerating the separation of the parts which mortify is by preventing the inflammation which disturbs this process, (p. 126;) and that the slough itself, far from being injurious, is a useful protection to the subjacent parts; "as, in fact, a first dressing, which is only removed after ten or fifteen days." (P. 122.)

*Lacerated Wounds.* Here M. Josse also labours to prove the advantage of cold water as applied to gunshot or lacerated wounds generally. The practice of the best British surgeons, both military and civil, and the works of all the most approved authors, go strongly to support the propriety of this as a general principle, although no doubt there are many exceptions. The chief points on which questions will arise, are, the period to which it should be prolonged; the application proper more immediately to the wounded part; and the abandonment of leeches in favour of cold water as advocated by the author. He states that, when cold water is applied directly after the injury, before reaction has taken place, and where it can be maintained with energy proportionate to the occasion, the phenomena of reaction will be prevented, (p. 142;) that heat, pain, and swelling will be subdued, and consequently the sympathetic fever will not take place; but when the cold water has not been applied before the development of the inflammatory symptoms, they will still be conquered by its efficient use, (p. 144;) and that reorganization takes place most favorably under its employment. After giving seven cases in which the benefits of this mode of practice are strongly exemplified, M. Josse sums up by stating that

"1st. When the action of a traumatic cause is not in itself sufficiently powerful nor assails organs of sufficient importance to cause immediate death, or render the continuance of the vital actions impossible, partial or general death only ensues from the violence of the inflammatory reaction. 2d. If we master the inflammation, bones, laid bare, deprived of their periosteum, do not become necrosed; broken, they unite in a short time; cut, they cicatrize as soon as the other tissues; tendons denuded, exposed to the action of the air and foreign bodies, do not exfoliate, nor form adhesions to their sheaths, but preserve their mobility; contused wounds may unite by immediate adhesion; vast collections of effused fluids may be absorbed, tissues contused, broken down, may reorganize themselves, &c.; and, 3dly, however severe may be the mischief, one may, by means of affusions, sufficiently master the inflammatory reaction, to render recourse to immediate amputation unnecessary." (P. 170.)

These positions, thus strongly laid down, we are by no means disposed to deny altogether; but we think they are stated too strongly, and that they *may*, would in all be a better term than they *do*. We must express our own persuasion to be that, in constitutions sufficiently robust, the unceasing hemorrhage of leeches, is paramount to all other means; that cold may be applied to other parts of the limb or body, so as to accomplish all the objects of refrigeration, as a most important auxiliary; but that, to the actual wound, the best application is often a simple soft bread-and-water poultice, either tepid or cold. With respect to the continuance of the cold, on which the author so strongly insists, we may remark that, although it should be persisted in as long as it evidently eases, or is not painful, yet such changes occur in the state of the part, as render its continued use improper, and either tepid applications, (according to the author,) or hot fomentations, which in many cases are, we believe, of greater advantage, must be resorted to, and in some instances are preferable from the beginning.

The opinion of one of our highest surgical authorities, as expressed on a recent occasion, and which we were not aware of at the time the preceding observations were written, goes strongly to corroborate the modified views to which our own experience had conducted us, in opposition to the sweeping dogmas of M. Josse. In the second number of the Guy's Hospital Reports, Mr. Key, in treating of *severe injuries of the large joints,* makes the following observations on the relative value of cold and hot applications:

"The expectation that inflammation will follow these accidents, leads to a practice that, in many instances, rather tends to impede convalescence than to assist nature's restorative efforts. The ordinary practice is that of applying cold, and keeping the parts in a state of complete rest; two steps, which, though very effective in arresting inflammation, are decidedly injurious, as far as my observation has enabled me to judge, in all recent strains and other accidents to the soft parts about joints. If we judge from a patient's feelings,—and they will often form not a bad criterion,—warmth and moisture are the applications which these accidents call for in the first instance, and which also contribute most to the quick restoration of the joint. I would go the length of saying that warm applications, besides being more grateful to the feelings of the patient, are more effectual in warding off inflammation than evaporating or cold lotions. The former determine blood to the surface of the limb, and keep the skin in a freely perspiring state; thus preventing that passive congestion of the deeper textures of the joint which is the first step towards active inflammation, &c. (*Guy's Reports*, ii. p. 258.)

As connected with this subject the author adverts to the question of amputation, which, as above stated, he is sanguine enough to believe, whether primary or secondary, will by this mode of practice be rendered seldom necessary, (p. 145 to 156.) It cannot be doubted that judicious treatment will save many limbs which otherwise must be sacrificed, but we believe, in civil practice, there is little occasion to influence the minds of surgeons to preserve more than they at present attempt to do; and, in military practice, the circumstances of the moment must always have a very powerful influence on the decision : such, however, is the undoubted efficacy of cold water in arresting the progress of inflammation in these cases, that we cannot avoid expressing a wish (which we believe must be participated by those who have witnessed the spectacle presented by the field of a great battle,) that arrangements could be made in all cases to procure a sufficient supply for such wounded men as, from various causes, cannot for a considerable period be removed. To those whose field of practice is the Navy, the mode of treatment here recommended will be, under all circumstances, of easy application; and we would here express a hope that some of them may soon put its efficacy to the test of experience.

*Gangrene.* The objects of the author in this chapter appear to be to confirm M. Dupuytren's opinion that the gangrene so admirably described by Mr. Pott, and commonly denominated senile, is of an inflammatory nature, and is best treated by antiphlogistic means and cold water; and that amputation is advantageous in this and certain other conditions of gangrene, in which its propriety has not been commonly admitted.

We regret to see the author commence by giving an arbitrary signification to terms in common use; a custom, it is true, less frequent of late than the unnecessary introduction of new ones, but both, we conceive, are much to be condemned. It is a practice liable to create confusion and trouble in a science already abounding in difficulties, many beyond our control; and it may not diminish these, perhaps, to teach us that by gangrene is understood complete mortification, (p. 197,) much less that the difference between it and sphacelus should likewise be understood to be, that gangrene is the death of a portion, sphacelus of the "whole thickness" of the part affected, (p. 198.) But to proceed to matter of more real importance.

M. Josse gives two cases, which he considers as unequivocally proving the existence of an inflammatory condition of the arteries in gangrena senilis, which disease he supposes to arise solely from an actual inflammation of these vessels commencing in the capillaries and propagated to the trunks, (p. 226.) The appearances on examination were, in the first, as follows: A great part of the foot was deeply disorganized; the tissues confounded, and not recognizable; the muscles of the sole retained their physical characters; the integuments and cellular membrane of the lower part of the leg were infiltrated with yellow fluid; the skin continued to be affected in the same way as far as the point of amputation; it was impossible to judge of the condition of the small arteries, such as the digital, but the larger were difficult to separate, red, and as it were infiltrated with blood externally; internally, extensive spots, in some places of a deeper, in others of a brighter red, covered nearly the whole of the tunics; this colour neither disappeared by washing nor friction. The parietes were very thick, and similar in colour and consistence to fibro-cartilage, the caliber of the anterior tibial was nearly obliterated, of the posterior in a less degree. These appearances diminished in approaching the point of section, but as they were still evident they must have extended into the parts above that point, (p. 211.) Such is a brief abstract of the appearances recorded in the first case, where the history accords with those commonly understood as characteristic of G. senilis, and where the limb was amputated with success. The patient, it may be observed, was only fifty.

The second case is an interesting one, inasmuch as the cause appears to have been the rupture of the femoral artery; but the phenomena much more resembled those of G. senilis, than those usually consequent on rupture of large arteries, so well described by Guthrie and Larrey. The following are the chief circumstances.

"A robust young man, æt. twenty-eight, received a kick from a horse in the right groin, followed by the usual symptoms of a most severe contusion in the part injured; and the great toe was, on the following day, cold, insensible, and discoloured; from which state it did not recover. Eight days afterwards, the patient feeling little inconvenience, walked to the house of one of his relations, and, in so doing, struck lightly against a stone, this was immediately followed by pain so severe that he was obliged to be carried home. The pain continued, accompanied by fever; the toe became black and shrunk; the other toes successively lost their sensibility and heat, changed their colour, and became shrunk; burning pains followed, accompanied by tetanic spasms. The sphacelus had invaded a large part of the foot, and was attended with great constitutional disturbance at the time the patient was seen by M. Josse, fifty days after the accident, and amputation was immediately recommended by him. As the disease was now making rapid progress, it was consented to two days afterwards, and performed at the usual distance below the knee. The principal arteries did not bleed; their parietes were thickened, their canal nearly obliterated, and a few drops of dark-coloured blood only escaped, (p. 216.) The severed portion presented 'perfectly similar' appearances to those of the first case."

Such is a very brief abstract of these two cases, which are narrated at great length.

Now, there can be little doubt that the arteries were inflamed in these cases: but, with reference to this point, is it clear that in other, it may be in all inflammations, whether tending to gangrene or not, the arteries of the part actually inflamed, may not be themselves more or less inflamed, either partly as cause or effect? In these cases narrated, however, the altered condition of the arteries was very great, and prevailed far above the gangrened part, most probably from extension of the diseased action along their tunics; and, as in every description of gangrene, the vessels are particularly affected, this circumstance may not only in itself prove a cause powerfully disposing to mortification alone, but of that spreading in the way it does. Further facts are wanting to throw light upon this subject: the cases related by M. Dupuytren, by Dr. Graves and Stokes, and these by the present author, all possess a degree of interest connected with it which fully warrants us in dwelling upon them.

With reference to the treatment, we must confess that, in cases occurring in advanced life, where the circulation is universally tardy, the vessels more or less diseased, the powers of maintaining heat feeble; we should be reluctant to apply actual cold or active antiphlogistic means, as M. Dupuytren and our author have recommended; but further experience is required on this point. As to the question of amputation, we have seen enough to convince us of its propriety in many instances; and we are now speaking of that form of the disease described by Mr. Pott.

M. Josse extends his remarks on the subject of amputation to gangrene generally, and we find him laying down the following positions: That inflammation produces further inflammation, (p. 227,) and that gangrene also, when commenced, proves a cause of more inflammation, and, by consequence, of its own further extension, (p. 239;) facts which there can be little reason to doubt, but which, we may remark, he is not the first to maintain. He proceeds to say, that, upon this principle, it may be advanced that amputation is proper when mortification *is spreading*, under the following circumstances:—(When it is *slow*.) 1st. When ligatures or other causes have obstructed the circulation, (a point now well established, if the cause of obstruction is in the limb;) 2d, when it has occurred without any known cause. Here also, he says, we may amputate without fear: " you will doubtless remove the cause together with the evil," (p. 241.) Upon this point, however, we apprehend, others may hesitate a little; but it is right to say that M. Josse founds his arguments in these cases on a pathological principle, which may be just, but which he rather assumes than proves,—namely, that, in *slow* mortification, the collateral circulation (of which the slow progress is an effect, and consequently a proof of its existence,) is perfect; but that, when it is *rapid*, the contrary is the case, (p. 239,) and amputation will not succeed. The observation is worthy of attention, though erroneous as regards sphacelus senilis; but we are bound to add, that, to our belief, the division into slow and rapid, without reference to cause or nature, is too arbitrary, and will not suffice: that which is slow to-day may become alarmingly rapid tomorrow; nor will that of dry and moist be more conclusive; for a gangrene may begin as the former, and in its progress assume the nature of the latter. To determine the point of amputation, it is necessary to ascertain with more precision the nature and the causes of the particular species of mortification under consideration, and to determine how far the latter is connected with the state of the general system, or otherwise; and, if it is, how far that may be remediable; also bearing in mind that, where the cause is originally local, the general system may *become* completely implicated in the progress, and that, to anticipate that implication, is often one of the most material points in surgery.

M. Josse concludes this chapter with the details of a curious and interesting case, which we regret our limits will not permit us to give more largely. A young man wounded his anterior tibial artery; an aneurism followed. The femoral was tied; and hemorrhage occurred when the ligature came away on the seventeenth day. It was not deemed advisable to tie it again at that point, or above, or even the external iliac, for reasons it would be too long to detail, and not quite conclusive. Under these circumstances pressure was adopted, first direct, then on the artery above, by means of assistants, who relieved each other *for twenty consecutive days and nights.* This proved effective, and the result was, that the femoral healed. The aneurism of the tibial was subsequently cured by laying open the sac, and securing the extremities of that vessel.

We now return to the third chapter, which is *on the Opening of Abscesses.* To this point, however, it is not confined; we find him discussing, in the first place, the proper applications to be used, and, in the second, the propriety of opening them, and the period and manner of doing so.

With reference to the former subject, *the applications to abscesses,* M. Josse is here, as elsewhere, very earnest in his condemnation of poultices, to which he denies the quality of being emollient: on the contrary, he charges them with increasing the pain and irritation, and the quantity of matter, (p. 95,) which, in itself, he says, becomes a new irritant, acting on the inflammation which produced it, (p. 97,) causing a pressure or strangulation, so to say, of the parietes; while he extols the use of cold water, as well after as before matter has been formed, and as well after as before openings have been made.

To persons accustomed to the frequent use of poultices, such practice will appear a little revolting, and there can be no question that our author is far too sweeping in his condemnation of one mode, and in commendation of the other: still we apprehend there is some room for his remarks; and, if he had discriminated with more accuracy the description of cases in which the one or the other plan of practice is preferable, he would have done good service to surgery. In many cases of facial, thecal, or cellular inflammation or erysipelas, where the parts want tone and adhesive action,—in short, in a large proportion of spreading inflammations,—poultices are often very injurious when matter is formed, particularly in the advanced stages, after openings have been made by nature or by art; and lotions, whether actually cold or not, whether the pure element alone or combined with medicines, will often be very preferable. On the other hand, who, having ever felt the effects of a poultice in phlegmon or in boil, would allow the accusation to be just, that it increases pain? It is not possible to see that process of relaxation of the skin which in such cases occur, and which Mr. Hunter has so well described, and allow that tension and strangulation, as M. Josse has stated, are the effects. Again, although, in spreading inflammations, the matter does become a source of fresh irritation to the surrounding parts in phlegmon, its secretion gives the most palpable relief to the symptoms, until it approaches near the surface. Here, as throughout, our author's remarks would have been just, if the discrimination had been so.

With respect to the second point, *on opening abscesses,* we find M. Josse advocating the following doctrines: 1st. That all acute abscesses should be opened, and with a cutting instrument, (p. 98;) 2d, that small openings are, in all cases, better than large ones, (p. 99 et seq.;) and, 3dly, that abscesses of a scrofulous character and buboes should be opened early, with small incisions. Now, we cannot say that we are persuaded of the justice of the first position to its full extent. We are most friendly to the opening, and the early opening, of spreading abscesses of every description; but the canon should not be absolute in phlegmon and some other limited abscesses, which are often better left to nature. The next position is still more open to objection. He argues that small incisions are preferable, because they give less pain, (but the difference is trifling;) because they leave smaller marks, (but this may be disregarded in every case but one;) because the incisions are new points of irritation, causing an increase of the secretion of pus in the cavity, and retarding the cure, (p. 101.) Here, we apprehend, M. Josse has overlooked the difference between acute and chronic abscesses: in the former, openings, when proper, and judiciously, we may say freely made, remarkably relieve, and the processes which ensue tend to immediate restoration: in the latter, on the contrary, as has been long since stated by Mr. Hunter, an opening, whether natural or artificial, no sooner takes place, than a new mode of inflammation commences on the parietes, often with very fatal effects. It was to obviate this that Mr. Abernethy advocated the plan of puncturing and immediately closing such collections; and, to lessen the degree of mischief, that Sir B. Brodie, we believe, recommends the anticipating a very large formation by an artificial opening, which will also evacuate it more in the natural mode, if effected by caustic, than by the knife. In these cases, it is agreeable to observation that such openings are better than large incisions, although we think it very doubtful whether the admission of air, to which M. Josse has briefly alluded, (p. 104,) is an essential cause of the phenomenon.

Again, M. Josse states, as a ground of objection to large incisions, that the edges gape, and that the cicatrices adhering to the subjacent parts impede the future functions of the member, especially over joints, (p. 104.) This point demands some enquiry. It is undoubtedly true that, in many cases where large incisions are made, such consequences are observed; but are they fairly attributable to the practice? We believe the truth to be this, that, in the cases where these results take place, the condition of the parts is such that, whether incisions are made or not, the skin and cellular membrane slough, ulcerate, or break down in suppuration, and thence the parts become fixed, and never perfectly regain their mobility; and this would happen if nature made the opening instead of art, with this difference, that the destruction would be vastly more extensive in the latter case, and the functions of the part consequently impaired to a greater degree, if not wholly destroyed. It is an advantage, however, in such cases, in favour of early openings, that they need not always be made very large; and we will concede to M. Josse that that integument will hold better together, if two or three small incisions are made near each other, than one large one, provided they sufficiently discharge the matter, and provided the disease has not gone so far as to terminate in the destructive processes alluded to above.

On the third point, so far as relates to the advantage of early openings for scrofulous abscesses, we apprehend that no one will doubt it when they occur in the face and neck; but as to buboes, which he also thinks should have an early and small opening, to avoid a discreditable mark, it so seldom happens that these are submitted to any but the friendly eye of a surgeon, that we can hardly think it need influence our plan of treatment.

One of the most valuable parts of the present work is the eighth chapter, which relates to the period and manner of *Dressing Wounds.*

It appears that, in 1815, M. Josse, sen. was appointed to the chief care of the wounded soldiers brought to Amiens, after the celebrated but short campaign of that year. Many of these had their wounds still covered with the same dressings which were placed upon them subsequent to the battle; and, in the midst of all the filth and stench, he was surprised to see how free from inflammation and how far advanced towards healing they were. On this observation he founded his future practice, and, being the surgeon of an hospital as large as many of the metropolis, he had afterwards ample opportunities of ascertaining its value; and his son informs us that the result has been decidedly in favour of postponing to a late period the second dressing, and of avoiding the subsequent renewal as much as possible. He contends that the natural processes are unnecessarily interfered with by removing the applications as early as the fourth or fifth day; that, at the period stated, the processes of reparation are in full play; that the suppuration is not as yet completely established, nor does this occur before the eighth, or from that to the fifteenth day, (p. 196;) that, to drag off the dressings at the early period stated, adhering as they do to the surface, is to inflict great injury on the parts, which previously were in no degree inflamed, and that the consequence is the production of very serious inflammation with its concomitants, phlebitis, tetanus, gangrene, &c., and that often sympathetic fever, which before scarcely existed, is excited by it; this fever itself tending still further to prevent the restorative actions, which alone go on well in a state of calm. A wound shielded by its dressings, and covered with its natural defence, pus, is, he says, in the condition of an abscess not yet opened, where all is quiet: if the pus is absorbed, the surface which takes it up renders its qualities innoxious: in such a wound, while a quantity of matter is formed, a certain quantity is also absorbed without danger, as in a common abscess; while it is only when it is taken into the vessels without change, that it induces the serious mischief so often observed, (p. 181.) These are the chief points supported in the present chapter, and, with limitations, we are disposed to acknowledge their justice.

We think M. Josse goes too far in laying down the minimum period so absolutely. A wound, under some circumstances, will as much require dressing on the third as another may on the twelfth day: thus, in very hot weather,—in unhealthy seasons,—in persons who have suffered amputation on account of diseases attended with large previous suppuration, such as scrofulous joints, compound fractures, &c., the suppurative diathesis being already established, all new wounds will run readily into that process, and require early dressings. In cases where much blood has been retained in the wound and is broken down, or where other foreign matters have lodged in it; where pus is not provided with a proper outlet when formed, or in stumps injudiciously united: in all these cases, and in many others, it is, we conceive, highly injurious to retain matters pent up which are acting as poisons on the part, and exciting most disastrous sympathy of the constitution, leading to that very destructive inflammation which our author so much deprecates; but there should be a *reason* for dressing, and we quite agree with M. Josse that it should be deferred till required; that we should never dress on the fifth or on any other day because the rules of art have so decreed, but, like his father, should feel and examine the stump or wound, as to whether it is tender, painful, will bear pressure, or the reverse; and, if its state is favorable, not change the dressings merely because they are offensive, or because the accustomed period has arrived. Neither, when the dressings have been once changed, is it right to remove them again after twenty-four hours, if it can possibly be avoided; for, as he says, it is not unusual to see the fever reappear, and continue for some hours, after every dressing, (p. 180.) In one respect we think M. Josse has been guilty of a serious omission; namely, in not sufficiently enforcing the propriety of dressing all wounds likely to suppurate, in such a way as to allow every egress for the pus, without disturbing that portion of the apparatus which maintains the parts in a fixed position, (we do not mean the edges in approximation:) it is the breaking down the new adhesions in the deep parts of the wound, loosening the recently agglutinated muscles and other tissues, which induces the necessity of deep suppuration to effect the process of restoration, and proves so exceedingly prejudicial; not the mere contact of air on the surface of such a wound, the apprehension of which has been magnified, in our opinion, to an unnecessary extent; for how could the wounds of all animals, other than man, be healed, if such were the prejudicial effects of air? M. Josse strongly enforces the propriety of applying cold water in all these cases, as might be naturally supposed; and he adduces the example of wounds on the cheeks, &c. to show that the constant application of moisture is not unfavorable to the rapidity with which the processes of restoration take place. We are bound to add, that the results of the mode of treatment adopted by M. Josse have been highly satisfactory; for he states (p. 187,) that, although the Hôtel Dieu at Amiens is very unfavorably situated, they do not now lose one amputation in twenty, and that phlebitis after it is unknown.

Here it may be right to sum up briefly the points which appear most worthy of approbation, or otherwise, in the preceding part of this essay; stating that, in advocating the employment of cold, whether in erysipelas or phlegmon, in mechanical injuries, or those from high temperature, it is quite clear that M. Josse brings forward no new practice, nor indeed does he claim to do so: the novelty consists, 1st, in the universality of the application; 2dly, the energy with which he employs it; and, 3dly, its duration, which, it will be observed, is commonly through all the stages of the disease. It is unnecessary to recapitulate the objections to be urged on the first head: the principle is evidently pushed too far; and while, on the one hand, it may be readily conceded that, in the early stages of most lacerated wounds, in thecal inflammations, through every stage, perhaps, in the more active forms of erysipelas, and in various cases of injuries from high temperature, the treatment by cold is the preferable one. On the other hand, there are many varieties of erysipelas in which the character of the inflammation is feeble or where the external affection is the least part of the disease, to which cold is very inapplicable; the same may be said of cellular inflammation, in which it has rarely been found to answer; and we may add, as a more general position, that, while it is often a most suitable remedy for spreading inflammations, it rarely is so for those of a limited nature, as, for instance, in boil and the various species of carbuncular abscess, to which ordinary glandular inflammation and simple phlegmon may be added. In inflammations of this description, its effects on the constitution are often prejudicial; and, with reference to the part itself, it rather seems to suspend the actions going on than to cure them, as (although long continued) these return with their former violence when it is left off: in such cases, cold will, if the expression may be used, render an acute inflammation chronic for a time. Of this, as of all the other remedies, internal as well as external, which have been confidently recommended as sure of general success in forms of disease having many varieties, it may be concluded, when the advocate is a man of talent and experience, that there must be considerable truth in the statement, but that it wants the limitations which the varying nature of disease always presupposes. The advantages which we believe are to be gained from the consideration of this part of the author's essay are chiefly to be derived from the second head; and we must again repeat our full conviction, that the important results which are capable of being obtained from the use of cold, when it is the appropriate remedy, are often lost from its inefficient application, and that nothing can be more judicious than the rules which he has laid down upon this point.

The eighth chapter contains the description of an apparatus for fractures of the neck and superior part of the thighbone, adapted also to the treatment of spontaneous luxations of that member. With regard to the former subject, it could hardly be expected that any very novel contrivance should be proposed, after the numerous endeavours which we have in our own time witnessed to remedy this accident. In point of fact, the present apparatus is a combination of many: thus, we have the long splint of Dessault, made very solid, and with certain modifications, to fix the limb; the aperture to provide for the discharge of the fæces, which constitutes so useful a part of Earle's bed, placed in a moveable frame, which more immediately supports the patient's body, by means of a strong web extended across it, and capable of being easily raised on four uprights, and thus not only affording due facility to the operation of elevating the patient, but rendering it practicable to keep the bed beneath constantly clean and duly arranged. On the bed he ordinarily rests, although the web which more immediately receives the body is interposed, and wholly supports it when it is desirable to raise it. So far there is nothing particularly novel in the plan; but the author further proposes a method of perpetual extension, which he produces in a mode not hitherto resorted to; for, having fixed the foot to the lower transverse limb of the long splint, he then, by means of two moveable side pieces placed beneath and at an angle with the level of the bed frame, calculated to act on the moveable frame above described, and capable of being adjusted to any angle by two sliding wedges, he raises the feet, and gives the surface on which the patient reposes an inclination greater or less, as may be desired, towards the head; by which, of course, the weight of the superior part of the body may be made to operate a continual but gentle extension on the upper portion of the fractured bone, the lower being retained immoveably by its attachment to the lower end of the splint, as above described. An inner splint is also applied along the inside of the limb as far as the foot, to steady it, and a small one in front of the thigh. This we believe to be a tolerably correct though very brief account of the apparatus in question: we say very brief, for the author's description extends over not less than twenty-one pages, and is moreover illustrated by eight plates.

M. Josse informs us that this plan has been employed in numerous cases of fracture, and been found to answer perfectly; he also states that no inconvenience arises from the depending position of the upper part of the patient's body. (P. 279.) We have ourselves seen great advantage derived from the same principle applied in the opposite direction,—namely, by adding weight to the limb through the intervention of a pulley, and fixing the upper part of the body, which is laid on a plane, inclined from the head downwards. Both plans will tend to accomplish the same purpose. Of the superiority of that proposed by the author we cannot, of course, judge, until it has been tried in this country; but we should conceive the inclination of the head downwards would prove unpleasant, if not injurious. His statement of the principal objects which are attained by it, it may be right to give.

"The muscles (he says,) passing over the fracture need not be compressed; the extensive and counter-extensive forces are distributed over the largest possible surface; the extension is absolutely in the direction of the axis of the limb; the extensive force is gradual, and easily regulated; the parts are sufficiently protected from the pressure of the straps and bandages; the rotation of the limb is prevented; the pelvis is comprehended in the apparatus; and, lastly, the apparatus itself is simple, easy to construct and apply, and little expensive." (P. 279.)

And he adds that, if care is taken that the moveable frame, the web of which more immediately supports the patient's body, should always rest evenly on the mattress, and that be daily adjusted, he may lie for months without galling or inconvenience; and, from this contrivance, may be lifted off the bed, and moved from place to place, or even be carried into the open air, if desirable.

So far with respect to fractures: but the title of the chapter is on the "Cure of Spontaneous Luxations;" and the author, in point of fact, employs the same apparatus for cases of luxated hip from disease, of which however he relates only two, in both of which benefit appears to have been derived from it. One, a little boy, who died of cerebral inflammation before the luxation was quite well, but at a time when he had quitted his bed; the other, a girl, about sixteen, who recovered perfectly. It appears that, in both, the limbs were much shortened (about four inches,) when the treatment commenced, and contracted: nevertheless, by management, the apparatus was usefully applied. The author also proposes its employment in cases where no luxation has yet taken place, to give the joint that perfect rest which, he says, cannot be otherwise obtained; but, as he makes no allusion to Mr. Earle's bed, we must suppose that he is not fully aware of its advantages in this respect.

At first sight, any measure of extension appears harsh; but, on the one hand, it will be perceived that the degree of force may be applied in the most gradual and gentle manner, and, on the other, it may be stated that in many cases of other diseased joints, particularly of the knee, the suffering produced by the constant contraction of the muscles acting upon them constitutes a great aggravation of the disease which it also probably increases; and thus gently overcoming this contraction, often affords very great relief. This so far tends to confirm the propriety of the author's practice in cases where the bone has not quitted its socket: when it has, the case is very different; it may be that in some its restoration will still be a benefit, but there are others in which it may be doubted whether it would be desirable, if capable of being accomplished. The parts of a joint, admirably suited for each other in health, may become a source of torture when applied to each other roughened by caries, and hence both the head of the bone and the socket may be more advantageously circumstanced, when the former has escaped from the latter, and both are in contact with soft parts only. We have seen many cases which have appeared to amend from the moment the bone has ceased to be bound down in its cavity, whether of hip or shoulder: but, be this as it may, we are willing to believe that, when it becomes a question of obtaining a *new* socket in these cases, that cannot well be situated more advantageously than in the neighbourhood and direction of the original, and that this apparatus may prove advantageous in determining its direction. To these remarks we are bound to add, that the application of this method must, in a considerable degree, obstruct the useful employment of local remedies; and that, in many instances, no management whatever will, we are persuaded, enable the surgeon to adapt it to the limb.

The author introduces in the sequel a disquisition on the advantages of excision of the end of the tibia, in cases of compound luxation of the ancle; and this constitutes the ninth and last chapter. He gives several interesting cases, in which the result of this practice appears to have been decidedly beneficial; but, although the subject is one of confessed importance, yet, as it is a point only to be determined by experience, and the cases M. Josse narrates are long, we feel that our limits will not allow us to give them. He claims the credit of novelty for this mode of treatment, inasmuch as it has been adopted hitherto only in cases where necrosis, &c. has occurred, or from necessity, in cases of luxation where the reduction of the bone was otherwise impracticable. He employs it as a *primitive* operation, to prevent the ill consequences which result from this accident. (P. 303, *et alibi.*)

We must now state in few words our opinion of this work. It embraces the consideration of many most important points in surgery, and these, it must be confessed, still far from being settled. The question of cold or warm applications in different inflammations, of the mode, the degree, or the duration of the former, if employed,—has hardly received hitherto the deliberate attention it deserves. The proper application of dressings, although the subject of several interesting memoirs recently published—and we particularly allude to Mr. Earle's on dressing injuries from heat, Mr. Macfadzen's mode of water dressing, Mr. Higginbottom's by caustic, and Mr. Stafford's, (all, as it appears to us, tending to the same point, namely, that of allowing every facility to nature to perform her own work, merely giving her protection,)—may receive further illustration from the present author. His proposal for the treatment of fractures of the thigh, not by a fixed power of extension, but by a force constantly acting, is one deserving of much attention. The last chapter we have alluded to also contains many valuable facts, and the principle it moots is of importance. Take this work altogether, it possesses considerable value, although it rarely happens that any one pushes his opinions to such an extreme length as our author does. It could have been wished that his details had been more condensed; a quality which, in these days of multiplied publications, is of the very first merit. His acquaintance with the literature of other countries we have to mention with just satisfaction; but we cannot at the same time help adding, that to us it appears most extraordinary that our neighbours should so often transform the names of British, and we may add German authors. Barbarous as some of them may perhaps be, they come out of the Gallic press with no improvement and cannot always be known. Klaterbrunner, it is true, cannot well be mistaken; but in With and Gook (p. 303,) it is hard to recognize the respected names of White and Gooch. It might be unreasonable to expect that they would correctly execute the difficult task of pronouncing our somewhat uncouth names, but we might surely expect that print would set the matter right, and give us the pleasure of seeing our compatriots quoted in all their just lineaments and proportions.

---

